# Trends in depression & anxiety symptom severity among mental health service attendees during the COVID-19 pandemic

**DOI:** 10.1016/j.jad.2021.04.020

**Published:** 2021-06-15

**Authors:** Rob Saunders, Joshua E.J. Buckman, Judy Leibowitz, John Cape, Stephen Pilling

**Affiliations:** a Centre for Outcomes and Research Effectiveness, Research Department of Clinical, Educational and Health Psychology, University College London, Gower Street, London, UK; b iCope – Camden and Islington Psychological Therapies Services, Camden & Islington NHS Foundation Trust, London, UK; c Camden & Islington NHS Foundation Trust, St Pancras Hospital, 4 St Pancras Way, London, UK

## Abstract

**Background:**

General population surveys have shown that some groups, particularly young women, experienced increased distress during nationally mandated restrictions to control the spread of COVID-19. However, there has been limited research on such trends among people with pre-existing mental health conditions, leaving mental health services ill equipped to plan for current and future lockdowns.

**Methods:**

Mean weekly scores on the GAD-7 and PHQ-9 between 01/01/2020-22/06/2020 (n=9,538 individuals) for all patients of two psychological treatment services (Improving Access to Psychological Therapies) in London, were compared to mean weekly scores from the same time periods in 2017-2019 (n=37,849). The proportion of scores which were above the clinical thresholds for ‘caseness’ each week were compared, and scores between groups based on gender, age group, and ethnicity, were also compared.

**Results:**

Confirmed community transmission in the UK (26/02/2020-03/03/2020) and the announcement of the national ‘lockdown’ (23/03/2020) were associated with significant increases in anxiety symptom scores. ‘Lockdown’ was associated with a decrease in depression scores. These changes were not maintained during lockdown. Significant increases in depression and anxiety were observed at week 23, as restrictions were eased.

**Limitations:**

This was an exploratory analysis in two services only. Residual confounding and selection biases cannot be ruled out.

**Conclusions:**

Differences in the weekly average symptom scores were short-term; they did not continue throughout ‘lockdown’ as might have been expected, except among older people. Replication of this study in other settings and investigating the potential benefits of more regular reviews or more intensive treatments for at-risk groups, are warranted.

## 1 Introduction

COVID-19 has had an unprecedented impact on healthcare services worldwide. The need for mental health treatment is anticipated to rise, as is distress in those with existing mental disorders (Luykx et al., 2020). Rises may be associated with fear and uncertainty about COVID-19 as well as consequences of governmental responses to the pandemic (‘lockdown’), including associated risks of loneliness, isolation, and financial pressures (Woolhandler and Himmelstein, 2020). For services and clinicians referring or treating patients with depression or anxiety disorders, understanding the impact of the pandemic on symptomatology is important in treatment planning and the clinical management of their conditions.

General population surveys have shown that whilst the majority of people's mental health appears unaffected in any significant way, some groups, particularly young women experienced increased distress during ‘lockdown’ (Fancourt et al., 2020; Saunders et al., 2021; Shevlin et al., 2020). However, there has been limited research on such trends among people with pre-existing mental health conditions, especially those in contact with services. One of the very few studies reported that 21% of hospital outpatients experienced a deterioration in their mental health condition related to the pandemic (Zhou et al., 2020), but we could find no studies of patients attending high-volume services in primary care or community settings. As many countries face further periods of government mandated ‘lockdowns’ in 2021, a better understanding of how this might impact patients’ mental health could inform service planning to mitigate the deleterious effects and support clinicians working with patients impacted by COVID-19. The current study explored trends in self-reported depression and anxiety symptoms for those attending UK primary care and community-based psychological treatment services each week during the first half of 2020 compared to average weekly scores over the three preceding years to track changes during the COVID-19 pandemic.

## 2 Method

### 2.1 Participants and measures

All recorded scores on the Generalized Anxiety Disorder scale (GAD-7: a seven-item screening measure for symptoms of generalised anxiety) (Spitzer et al., 2006) and the Patient Health Questionnaire (PHQ-9: a nine-item screening measure for symptoms of depression) (Kroenke et al., 2001) from 01/01/2020 to 22/06/2020 (n=9,538 individuals) were extracted from electronic health records from Camden and Islington Improving Access to Psychological Therapies (IAPT) services (London, UK), alongside scores from the same time periods in 2017-2019 (n=37,849). These services provide evidence-based psychological treatments for depression and anxiety disorders as part of the UK National Health Service (Clark, 2018).

### 2.2 Data analysis

The mean GAD-7 and PHQ-9 score for each calendar week between 01/01/2020 until the 22/06/2020 were compared to the mean weekly scores from January to the third week in June across 2017, 2018 and 2019, combined. Differences between the means were compared using linear regression models controlling for age, gender, and ethnicity of the patient providing each score. We also compared the proportion of scores which were above the clinical thresholds for ‘caseness’ (≥10 on the PHQ-9 and ≥8 on the GAD-7) (NHS Digital, 2017); and compared scores between groups based on gender, age group, and ethnicity. These covariates were used as categorical variables with a dummy coded category for missing values to ensure all participants could be included in analyses (i.e. not lost due to list-wise deletion).

For the primary analyses, we included any PHQ-9 or GAD-7 score recorded by or sent to the services, regardless of whether they were for initial assessments, treatment sessions or final reviews, each week was treated as an independent wave of data collection. We employed no exclusion criteria on scores. Some patients will therefore have contributed to multiple weeks, but within person differences were not accounted for in analyses as the research question here related to the overall levels of distress for all patients attending at the two services each week during the study period. The number of referrals each week are also included for reference. Further analysis using only initial assessment (first contact) scores were also conducted. Analyses were conducted in Stata16 (Stata Press, 2019). For further methodological details, see Supplementary Materials.

### 2.3 Ethical considerations

This evaluation was completed as part of a wider service improvement project conducted in accordance with the procedures of the host institution and the NHS Trusts which operate the services (project reference: 00519-IAPT). NHS ethical approval was not required for this study (confirmed by the Health Research Authority July 2020, reference number 81/81).

## 3 Results

**Fig. 1:**
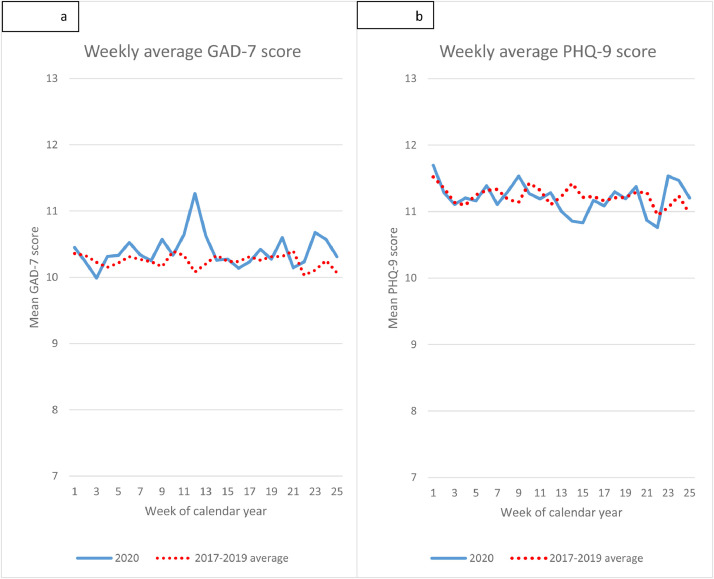
Average weekly GAD-7 (Fig. 1a) and PHQ-9 scores (Fig. 1b).

Descriptive statistics of the sample are provided in Supplementary eTable1. The average weekly GAD-7 and PHQ-9 scores in the first 25 weeks of 2020 and 2017-2019 combined are presented in Fig. 1a and b, with the mean weekly scores, beta coefficients (B), and 95% confidence intervals (CIs) are presented in Supplementary eTable2. Compared to the average weekly scores from the previous three years, there was no evidence for differences in GAD-7 2020 weekly averages until Week 9 (26/02/2020-03/03/2020; coefficient (B)=0.39, 95% confidence intervals(95%CI)= 0.01,0.78) before a spike at Week 12 (18/03/2020-24/03/2020; B(95%CI)= 1.15(0.74,1.57) and higher scores at Week 13 (B(95%CI)= 0.49(0.08,0.91)). These correspond to the first confirmed cases of COVID-19 in England (Week 9) and significant increases in deaths followed by the announcement of national lockdown by the government (Week 12). In comparison, there was no evidence of differences in weekly PHQ-9 scores until a decrease at Week 14, and therefore in the early weeks of ‘lockdown’ (B(95%CI)= -0.51(-0.99,-0.03)).

Average GAD-7 and PHQ-9 scores during the following weeks of ‘lockdown’ were similar to previous years, until a significant increase at Week 23 (03/06/2020-09/06/2020; GAD-7: B(95%CI)= 0.57(1.7-0.97); PHQ-9: B(95%CI)= 0.49(0.03,0.95)) which corresponds to the easing of ‘lockdown’; people returning to work and school. There was also a 75% decrease in referral numbers at Week 9 (n=579) compared to Week 12 (n=140) of 2020, which will have reduced the number of available symptom severity scores from weeks 12 onwards.

**Fig. 2:**
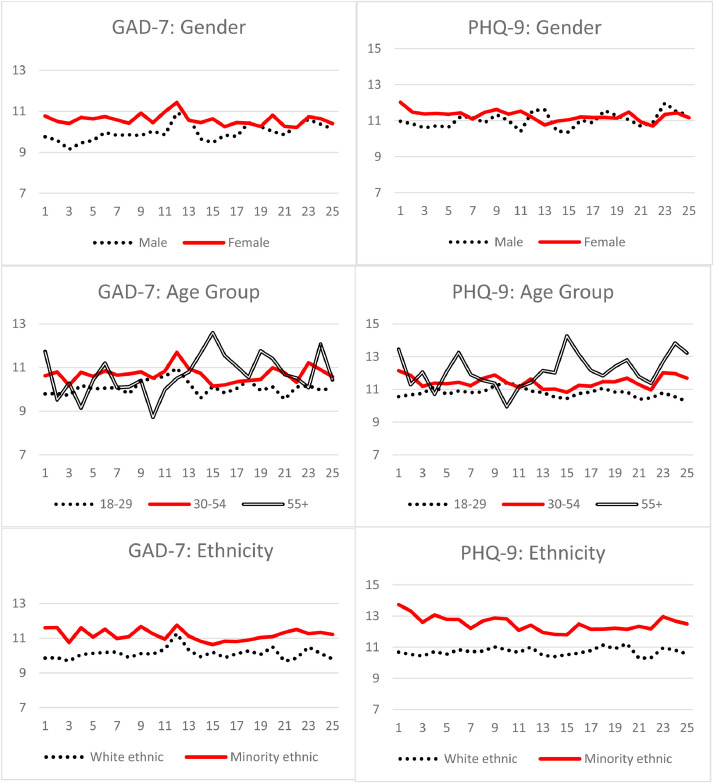
Average weekly GAD-7 and PHQ-9 scores by gender (top panel), age group (middle panel) and ethnic group (bottom panel).

The proportion of scores which were indicative of 'caseness' are presented in Supplementary eTable3 and eFigure1. The trends are very similar to the average weekly scores. There was evidence that there were significantly more scores above the clinical cut-off on the GAD-7 at week 12 and week 23, and that there were significantly fewer PHQ-9 scores above the cut-off at week 14, and more at week 23. Analysis of only the initial assessment (baseline) scores for all patients attending the services from Jan 2017 to June 2020 is presented in Supplementary eFigure2. Initial symptom severity scores did not appear to be higher in the lockdown period (April to May 2020), but they were slightly higher in the weeks post-lockdown. Further analysis of the 2020 scores alone indicated similar trends in GAD-7 scores between men and women, whereas PHQ-9 scores varied more between genders week-by-week during lockdown (Fig. 2). On average, younger patients reported lower scores, whereas older patients reported higher scores in the lockdown period, and patients from minority ethnic groups consistently scored higher than white ethnic patients (Fig. 2).

## 4 Discussion

This exploratory analysis highlighted the brief but significant spikes in generalised anxiety in mental health service attendees following the first confirmed cases of COVID-19 nationwide and announcements of both commencing and easing of ‘lockdown’. The increases following 'lockdown' might represent anxiety associated with change and uncertainty, including fears of contracting the virus and the impact of lockdown on personal finances or employment (Brooks et al., 2020; McGinty et al., 2020). Some of the increase in anxiety may also be associated with service-level changes including the necessary shift to delivering interventions remotely (Buckman et al., 2021). This change may have resulted in increased anxiety with patients already in the middle of treatment, however, for new patients, initial assessment scores were very slightly trending downwards in the years before and during lockdown, but appear to rising in line with other scores post-lockdown. This may be linked to the lower number of referrals during the lockdown, and therefore people delaying seeking support for mental health concerns, and suggests that monitoring the symptom severity of patients presenting to services would be of value for service planning. Interestingly, depression scores were observed to significantly decrease during the first weeks after ‘lockdown’ before returning to levels similar to the previous year, but then increased following the easing of restrictions. This might reflect the gain and then loss of enhanced ‘community spirit’ that was reported to have been experienced during ‘lockdown’, and rising financial pressures which may have become apparent as people returned to work or began looking for new jobs after ‘lockdown’ (Rutter, 2020).

It is noteworthy that differences in the weekly average symptom scores were short-term; they did not continue throughout ‘lockdown’ as might have been expected, except among older people. UK general population studies have suggested that average depression and anxiety scores were highest in the initial weeks of the pandemic before decreasing (Fancourt et al., 2020). However, a sub-group of individuals had high anxiety scores in the first weeks of the pandemic, which then decreased rapidly to levels observed in the general population within the first few weeks (Saunders et al., 2021), and may mirror the observed initial increases in anxiety observed in the current study.

This study included all weekly PHQ-9 and GAD-7 scores, and made no exclusions for the types of clinical appointments the patient had, but several potential confounders could not be controlled for, including information on personal experiences of COVID-19 or on domestic violence (Bhavsar et al., 2020; Brooks et al., 2020). The number of referrals dropped following national lockdown which resulted in fewer new patients providing data for the analysis from weeks 12 onwards, although the minimum number each week was over 500. That the increase in older people's GAD-7 and PHQ-9 scores was maintained during the lockdown in this sample, which differs from general population findings (Fancourt et al., 2020), might highlight a sub-group of patients at particularly high-risk of increased mental distress due to pandemic. This may also be due to changes in service delivery to remote treatments, with some suggestion that remote treatment via video-calling is associated with preferable outcomes compared to remote treatment over the telephone (Buckman et al., 2021), but is less accessible to older adults.

## 5 Limitations

We adjusted for a number of patient characteristics but residual confounding cannot be ruled out. The analysis here was not focussed on changes in the symptom scores of individual patients throughout the weeks of 2020, and as such, controlling for other personal characteristics might have introduced other biases. For example, we might have controlled for patient's presenting problems, but as these are typically recorded at the point patients enter treatment this would also have removed variance due to the stage of each person's care within the services and therefore would not have allowed us to answer the research question here. In addition, selection biases cannot be ruled out; referrals to the services fell during ‘lockdown’ resulting in fewer scores being recorded. Further, this was an exploratory analysis in two London services only, replication in other services and settings is needed before generalisable conclusions can be drawn.

## 6 Conclusions

The UK government mandated lockdown to control the COVID-19 pandemic appears to have led to higher levels of anxiety among attendees of two primary care and community based mental health services. This peaked and fell during lockdown for most patients, although older adults recorded GAD-7 scores that were consistently higher than pre-lockdown during the study period. The mental health of older adults that attend such services might be particularly affected during the pandemic, potentially linked to the move to remote treatment by services, and therefore further investigation of ways of reducing distress associated with COVID-19 in this group might improve the treatment experience of these individuals.

## Authors' contributions

JB and SP contributed to gaining funding for the current study. RS and JB contributed to the study design, literature search, conducted the analysis, and drafted the initial versions of the manuscript and appendix. JL contributed to the study design, data acquisition, data interpretation and final approval. JC contributed to the study design, analysis plan, data interpretation, drafting of the manuscript, and final approval. SP contributed to the study design, analysis plan, data interpretation, drafting of the manuscript, and final approval.

## Funding

This work was supported by the 10.13039/100010269Wellcome Trust (Grant Code 201292/Z/16/Z) and the 10.13039/501100000272National Institute for Health Research University College London Hospitals Biomedical Research Centre, University College London. None of these funders had any role in the study design, collection, analysis or interpretation of the data, writing the manuscript, or the decision to submit the paper for publication.

## Declaration of Competing Interest

None to declare.

## Appendix Supplementary materials

Image, image 1
