# Supplementary material for: Trends in depression & anxiety symptom severity among mental health service attendees during the COVID-19 pandemic
Source: J Affect Disord. 2021 Jun 15;289:105–9. doi: 10.1016/j.jad.2021.04.020 (PMC8374083; doi:10.1016/j.jad.2021.04.020)
Supplement: Supplementary file 1 [file mmc1.docx]

Supplementary methodological detail to:

Trends in depression & anxiety symptom severity among mental health service attendees during the COVID-19 pandemic

The data for the current study was provided by Camden and Islington (C&I) Improving Access to Psychological Therapies (IAPT) services. This dataset includes all self-reported depression and generalised anxiety symptom severity scores returned to the services from the 01/01/2017 to 22/06/2020. The dataset was extracted by the services on the 25/06/2020 and transferred to a secure server at the host institution on the same day. The PHQ-9 and GAD-7 scores used in this study were either completed by service users through secure online web portals or in collaboration with a clinician who entered the information onto the electronic healthcare record system. The services are mandated to collect routine outcome measurement data on depression and anxiety symptom severity, and therefore service users are expected to complete measures at initial assessments and at each treatment session or clinical contact. Requests to complete these measures are sent automatically through the electronic patient management system.

The descriptive statistics of the sample are provided in eTable 1 below. The main ‘presenting problem’ is used as a proxy for diagnosis in the services in order to match patients to evidence-based treatments for specific disorders. Problem descriptors were grouped into categories used in previous analyses of similar datasets, this included a small number of patients being classified as having a severe mental illness or ‘other’ problem for which there are no IAPT treatment protocols (Buckman et al., 2018; Saunders et al., 2019). As this dataset included all individuals, including referrals and patients who were eventually not taken on for treatment and briefly assessed only, a significant amount of individuals (45%) did not have presenting problems recorded as these are more typically recorded at the point a patient enters treatment and has completed a more thorough assessment of their needs.

**eTable1. Descriptive statistics of sample provided symptom scores**

| Variable | Category | N | % |
| --- | --- | --- | --- |
| Age | 18-29 | 20434 | 39.08% |
|  | 30-54 | 24400 | 46.66% |
|  | 55+ | 7330 | 14.02% |
|  | Missing | 126 | 0.24% |
| Gender | Male | 16482 | 31.52% |
|  | Female | 35581 | 68.05% |
|  | Missing | 227 | 0.43% |
| Ethnicity | White | 32052 | 61.30% |
|  | Mixed | 3476 | 6.65% |
|  | Asian | 4627 | 8.85% |
|  | Black | 4606 | 8.81% |
|  | Chinese | 796 | 1.52% |
|  | Other | 2555 | 4.89% |
|  | Missing | 4178 | 7.99% |
| LTC status‡ | No | 31999 | 61.20% |
|  | Yes | 14337 | 27.42% |
|  | Missing | 5954 | 11.39% |
| Presenting problem | Depression | 13686 | 26.17% |
|  | Mixed anxiety and depression | 961 | 1.84% |
|  | Generalised anxiety disorder (GAD) | 6269 | 11.99% |
|  | Obsessive-compulsive disorder (OCD) | 795 | 1.52% |
|  | Post-traumatic stress disorder (PTSD) | 1064 | 2.03% |
|  | Phobic anxiety & Panic | 3216 | 6.15% |
|  | Severe mental illness (SMI) | 87 | 0.17% |
|  | Other | 2941 | 5.62% |
|  | Missing | 23271 | 44.50% |
|  | | Mean | SD |
| Initial GAD-7 score* | | 12.27 | 5.37 |
| Initial PHQ-9 score* | | 13.64 | 6.35 |

Notes: * Initial scores are the first PHQ-9/GAD-7 scores available for individuals. ‡ LTC status = whether the patient self-reports having a long-term physical health condition or not.

**eTable2: Weekly number of referrals, average GAD-7 and PHQ-9 scores.**

| **Week of calendar year** | **Number of referrals** | | **GAD-7 Scores** | | | | | | | | | **PHQ-9 Scores** | | | | | | | | |
| --- | --- | --- | --- | --- | --- | --- | --- | --- | --- | --- | --- | --- | --- | --- | --- | --- | --- | --- | --- | --- |
|  | **2020** | **2017-2019** | 2017-2019 | | |  | 2020 | | |  |  | 2017-2019 | | |  | 2020 | | |  |  |
|  |  |  | N | Mean | SD |  | N | Mean | SD | B | 95% CIs | N | Mean | SD |  | N | Mean | SD | B | 95% CIs |
| **1** | 456 | 331 | 1887 | 10.36 | 5.72 |  | 648 | 10.45 | 5.57 | 0.04 | (-0.46;0.55) | 1887 | 11.52 | 6.54 |  | 649 | 11.70 | 6.68 | 0.14 | (-0.44;0.72) |
| **2** | 500 | 447 | 3538 | 10.33 | 5.58 |  | 1011 | 10.23 | 5.59 | -0.12 | (-0.51;0.27) | 3544 | 11.35 | 6.38 |  | 1011 | 11.28 | 6.27 | -0.08 | (-0.53;0.36) |
| **3** | 577 | 423 | 3460 | 10.23 | 5.66 |  | 1044 | 9.99 | 5.45 | -0.24 | (-0.63;0.14) | 3465 | 11.14 | 6.41 |  | 1044 | 11.11 | 6.29 | -0.06 | (-0.5;0.38) |
| **4** | 590 | 428 | 3644 | 10.15 | 5.53 |  | 1094 | 10.32 | 5.48 | 0.15 | (-0.22;0.52) | 3651 | 11.10 | 6.39 |  | 1094 | 11.20 | 6.27 | 0.08 | (-0.34;0.51) |
| **5** | 638 | 436 | 3510 | 10.22 | 5.56 |  | 1084 | 10.33 | 5.52 | 0.08 | (-0.3;0.46) | 3513 | 11.25 | 6.50 |  | 1086 | 11.16 | 6.33 | -0.12 | (-0.55;0.32) |
| **6** | 597 | 471 | 3417 | 10.31 | 5.54 |  | 1114 | 10.53 | 5.53 | 0.21 | (-0.17;0.58) | 3423 | 11.32 | 6.39 |  | 1114 | 11.39 | 6.50 | 0.07 | (-0.36;0.5) |
| **7** | 531 | 432 | 3303 | 10.27 | 5.65 |  | 1041 | 10.34 | 5.45 | 0.07 | (-0.32;0.46) | 3304 | 11.34 | 6.49 |  | 1042 | 11.11 | 6.24 | -0.23 | (-0.67;0.22) |
| **8** | 558 | 407 | 3407 | 10.24 | 5.55 |  | 949 | 10.25 | 5.44 | -0.01 | (-0.41;0.38) | 3416 | 11.18 | 6.36 |  | 953 | 11.31 | 6.23 | 0.09 | (-0.36;0.54) |
| **9** | 579 | 423 | 3309 | 10.16 | 5.61 |  | 1033 | 10.57 | 5.48 | 0.39 | (0;0.78) | 3315 | 11.14 | 6.53 |  | 1034 | 11.53 | 6.26 | 0.36 | (-0.09;0.82) |
| **10** | 474 | 450 | 3475 | 10.39 | 5.59 |  | 1002 | 10.34 | 5.43 | -0.08 | (-0.47;0.31) | 3480 | 11.43 | 6.42 |  | 999 | 11.27 | 6.40 | -0.19 | (-0.64;0.26) |
| **11** | 294 | 465 | 3521 | 10.33 | 5.59 |  | 908 | 10.64 | 5.62 | 0.34 | (-0.07;0.75) | 3525 | 11.32 | 6.48 |  | 908 | 11.19 | 6.44 | -0.08 | (-0.55;0.39) |
| **12** | 140 | 446 | 3525 | 10.08 | 5.53 |  | 840 | 11.26 | 5.53 | 1.15 | (0.74;1.57) | 3540 | 11.11 | 6.36 |  | 845 | 11.29 | 6.40 | 0.14 | (-0.34;0.62) |
| **13** | 155 | 401 | 2909 | 10.20 | 5.47 |  | 856 | 10.63 | 5.40 | 0.49 | (0.08;0.91) | 2911 | 11.23 | 6.38 |  | 859 | 11.00 | 6.27 | -0.13 | (-0.61;0.36) |
| **14** | 148 | 412 | 3032 | 10.33 | 5.55 |  | 893 | 10.26 | 5.26 | -0.02 | (-0.43;0.39) | 3035 | 11.43 | 6.54 |  | 893 | 10.86 | 6.15 | -0.51 | (-0.99;-0.03) |
| **15** | 133 | 406 | 3096 | 10.24 | 5.45 |  | 538 | 10.28 | 5.37 | 0.09 | (-0.41;0.59) | 3097 | 11.21 | 6.31 |  | 539 | 10.83 | 6.31 | -0.26 | (-0.84;0.31) |
| **16** | 171 | 329 | 2633 | 10.24 | 5.49 |  | 801 | 10.14 | 5.21 | -0.07 | (-0.5;0.36) | 2634 | 11.23 | 6.40 |  | 802 | 11.17 | 6.07 | -0.01 | (-0.51;0.49) |
| **17** | 209 | 462 | 3236 | 10.31 | 5.59 |  | 872 | 10.23 | 5.40 | -0.10 | (-0.51;0.32) | 3236 | 11.16 | 6.47 |  | 873 | 11.09 | 6.13 | -0.09 | (-0.57;0.39) |
| **18** | 251 | 402 | 3079 | 10.26 | 5.59 |  | 903 | 10.42 | 5.17 | 0.09 | (-0.32;0.5) | 3084 | 11.21 | 6.47 |  | 906 | 11.30 | 5.96 | 0.06 | (-0.42;0.53) |
| **19** | 231 | 446 | 3535 | 10.31 | 5.50 |  | 785 | 10.27 | 5.32 | -0.01 | (-0.44;0.41) | 3537 | 11.21 | 6.30 |  | 784 | 11.19 | 6.02 | 0.06 | (-0.43;0.55) |
| **20** | 237 | 473 | 3755 | 10.32 | 5.59 |  | 875 | 10.60 | 5.40 | 0.32 | (-0.08;0.73) | 3763 | 11.29 | 6.47 |  | 876 | 11.38 | 6.01 | 0.20 | (-0.27;0.67) |
| **21** | 198 | 423 | 3369 | 10.40 | 5.46 |  | 723 | 10.14 | 5.21 | -0.21 | (-0.65;0.22) | 3371 | 11.29 | 6.34 |  | 723 | 10.87 | 5.83 | -0.34 | (-0.85;0.16) |
| **22** | 241 | 392 | 2852 | 10.03 | 5.43 |  | 932 | 10.23 | 5.39 | 0.22 | (-0.18;0.62) | 2854 | 10.95 | 6.29 |  | 933 | 10.76 | 6.03 | -0.14 | (-0.61;0.32) |
| **23** | 290 | 409 | 3565 | 10.11 | 5.52 |  | 906 | 10.68 | 5.44 | 0.57 | (0.17;0.97) | 3568 | 11.06 | 6.37 |  | 908 | 11.53 | 6.22 | 0.49 | (0.03;0.95) |
| **24** | 304 | 429 | 3480 | 10.25 | 5.52 |  | 789 | 10.57 | 5.46 | 0.38 | (-0.04;0.81) | 3485 | 11.24 | 6.45 |  | 789 | 11.47 | 6.24 | 0.34 | (-0.16;0.83) |
| **25** | 250 | 399 | 3542 | 10.07 | 5.67 |  | 514 | 10.31 | 5.60 | 0.26 | (-0.27;0.78) | 3551 | 10.97 | 6.40 |  | 516 | 11.21 | 6.45 | 0.26 | (-0.33;0.85) |

Notes: N is the number of observations providing data each week. Beta-coefficients (B) and 95% confidence intervals (95% CIs) from linear regression models controlling for age, gender and ethnicity.

**eTable3: Weekly proportion scoring above clinical cut-offs on the GAD-7 and PHQ-9.**

| **Week of calendar year** | **GAD-7 Above Cut-off** | | | | | | | | **PHQ-9 Scores** | | | | | | | |
| --- | --- | --- | --- | --- | --- | --- | --- | --- | --- | --- | --- | --- | --- | --- | --- | --- |
|  | **2020** | | |  | **2017-2019** | | |  | **2020** | | |  | **2017-2019** | | |  |
|  | **N = Above cut off** | **Total N** | **%** |  | **N = Above cut off** | **Total N** | **%** | **p-value** | **N = Above cut off** | **Total N** | **%** |  | **N = Above cut off** | **Total N** | **%** | **p-value** |
| **1** | 419 | 648 | 65% |  | 1192 | 1887 | 63% | 0.496 | 369 | 649 | 57% |  | 1087 | 1887 | 58% | 0.740 |
| **2** | 630 | 1011 | 62% |  | 2228 | 3538 | 63% | 0.702 | 590 | 1011 | 58% |  | 2009 | 3544 | 57% | 0.344 |
| **3** | 642 | 1044 | 61% |  | 2167 | 3460 | 63% | 0.507 | 571 | 1044 | 55% |  | 1890 | 3465 | 55% | 0.933 |
| **4** | 707 | 1094 | 65% |  | 2269 | 3644 | 62% | 0.157 | 621 | 1094 | 57% |  | 2018 | 3651 | 55% | 0.384 |
| **5** | 687 | 1084 | 63% |  | 2172 | 3510 | 62% | 0.374 | 583 | 1086 | 54% |  | 1944 | 3513 | 55% | 0.338 |
| **6** | 728 | 1114 | 65% |  | 2157 | 3417 | 63% | 0.180 | 621 | 1114 | 56% |  | 1925 | 3423 | 56% | 0.774 |
| **7** | 661 | 1041 | 63% |  | 2072 | 3303 | 63% | 0.656 | 575 | 1042 | 55% |  | 1858 | 3304 | 56% | 0.551 |
| **8** | 588 | 949 | 62% |  | 2146 | 3407 | 63% | 0.562 | 538 | 953 | 56% |  | 1889 | 3416 | 55% | 0.526 |
| **9** | 679 | 1033 | 66% |  | 2064 | 3309 | 62% | 0.051 | 595 | 1034 | 58% |  | 1820 | 3315 | 55% | 0.136 |
| **10** | 628 | 1002 | 63% |  | 2235 | 3475 | 64% | 0.340 | 545 | 999 | 55% |  | 1962 | 3480 | 56% | 0.306 |
| **11** | 587 | 908 | 65% |  | 2222 | 3521 | 63% | 0.390 | 502 | 908 | 55% |  | 1996 | 3525 | 57% | 0.469 |
| **12** | 579 | 840 | 69% |  | 2185 | 3525 | 62% | <0.001 | 476 | 845 | 56% |  | 1937 | 3540 | 55% | 0.397 |
| **13** | 566 | 856 | 66% |  | 1834 | 2909 | 63% | 0.100 | 460 | 859 | 54% |  | 1624 | 2911 | 56% | 0.246 |
| **14** | 558 | 893 | 62% |  | 1948 | 3032 | 64% | 0.335 | 464 | 893 | 52% |  | 1708 | 3035 | 56% | 0.023 |
| **15** | 340 | 538 | 63% |  | 1963 | 3096 | 63% | 0.927 | 279 | 539 | 52% |  | 1724 | 3097 | 56% | 0.093 |
| **16** | 502 | 801 | 63% |  | 1665 | 2633 | 63% | 0.772 | 448 | 802 | 56% |  | 1452 | 2634 | 55% | 0.714 |
| **17** | 553 | 872 | 63% |  | 2023 | 3236 | 63% | 0.625 | 483 | 873 | 55% |  | 1780 | 3236 | 55% | 0.866 |
| **18** | 595 | 903 | 66% |  | 1933 | 3079 | 63% | 0.088 | 515 | 906 | 57% |  | 1703 | 3084 | 55% | 0.387 |
| **19** | 510 | 785 | 65% |  | 2226 | 3535 | 63% | 0.293 | 444 | 784 | 57% |  | 1974 | 3537 | 56% | 0.675 |
| **20** | 576 | 875 | 66% |  | 2341 | 3755 | 62% | 0.054 | 501 | 876 | 57% |  | 2111 | 3763 | 56% | 0.557 |
| **21** | 463 | 723 | 64% |  | 2185 | 3369 | 65% | 0.676 | 391 | 723 | 54% |  | 1893 | 3371 | 56% | 0.308 |
| **22** | 589 | 932 | 63% |  | 1758 | 2852 | 62% | 0.395 | 502 | 933 | 54% |  | 1531 | 2854 | 54% | 0.932 |
| **23** | 599 | 906 | 66% |  | 2190 | 3565 | 61% | 0.009 | 525 | 908 | 58% |  | 1931 | 3568 | 54% | 0.045 |
| **24** | 516 | 789 | 65% |  | 2197 | 3480 | 63% | 0.232 | 455 | 789 | 58% |  | 1924 | 3485 | 55% | 0.209 |
| **25** | 322 | 514 | 63% |  | 2183 | 3542 | 62% | 0.658 | 279 | 516 | 54% |  | 1916 | 3551 | 54% | 0.962 |

Note: N is the number of observations providing data each week. P-values from Chi-Square test of independence.

In addition to comparing average scores on both the GAD-7 and PHQ-9, we also compared the proportion of individuals scoring above the clinical threshold on both measures for each week in 2020 to the proportion over the previous three years. The cut-off used for the PHQ-9 was scores ≥10, and the cut-off on the GAD-7 was ≥8, these are the thresholds used by the services, all other IAPT services nationally, and those reported by the originators of each scale. The proportion of individuals above the clinical threshold on each measure is presented in eTable3 above, with the trends in the number of individuals meeting caseness presented in eFigure1a and 1b below.

eFigure1b

*eFigure1: The proportion of scores above clinical cut-offs on the GAD-7 (eFigure 1a) and the PHQ-9 (eFigure 1b) per week*

Further analysis explored the trends in initial (baseline) assessment GAD-7 and PHQ-9 scores for patients attending the services from January 2017 to June 2020. There has been a slightly decreasing trend in initial symptom severity scores, suggesting that overall patients are presenting with marginly lower scores over time, but considerable variation is observed per week. Scores for patients assessed in April and May 2020 did not appear observably higher, but those for June 2020 appear to be increasing slightly, as the number of referrals is also increasing.

*eFigure2: Average initial assessment (baseline) GAD-7 & PHQ-9 scores from Jan 2017 to June 2020.*

**References**

Buckman, J.E.J., Naismith, I., Saunders, R., Morrison, T., Linke, S., Leibowitz, J., Pilling, S., 2018. The Impact of Alcohol Use on Drop-out and Psychological Treatment Outcomes in Improving Access to Psychological Therapies Services: an Audit. Behav. Cogn. Psychother. 46, 513–527. https://doi.org/10.1017/S1352465817000819

Saunders, R., Buckman, J.E.J., Cape, J., Fearon, P., Leibowitz, J., Pilling, S., 2019. Trajectories of depression and anxiety symptom change during psychological therapy. J. Affect. Disord. 249. https://doi.org/10.1016/j.jad.2019.02.043
